# Cognitive outcomes following functional neurosurgery in refractory OCD patients: a systematic review

**DOI:** 10.1007/s10143-023-02037-w

**Published:** 2023-06-23

**Authors:** G. Laseca-Zaballa, G. Lubrini, J. A. Periañez, V. Simón-Martínez, M. Martín Bejarano, C. Torres-Díaz, N. Martínez Moreno, J. Álvarez-Linera, R. Martínez Álvarez, M. Ríos-Lago

**Affiliations:** 1 Department of Experimental Psychology, Cognitive Processes and Speech Therapy, Complutense University of Madrid, Madrid, Spain; 2 Department of Basic Psychology II, UNED, Madrid, Spain; 3 General Hospital of the Defense, Zaragoza, Spain; 4 Neuropsychology Service, Fidias Health & Sport, Cádiz, Spain; 5 Faculty of Medicine, University of Cádiz, Cádiz, Spain; 6 Department of Radiosurgery and Functional Neurosurgery, Ruber International Hospital, Madrid, Spain; 7 Department of Radiodiagnosis, Ruber International Hospital, Madrid, Spain; 8 Brain Damage Service, Beata Maria Ana Hospital, Madrid, Spain

**Keywords:** Capsulotomy, Cingulotomy, Cognition, Neuropsychology, Neurosurgery, Obsessive–compulsive disorder

## Abstract

Neurosurgery is a therapeutic option for patients with refractory obsessive–compulsive disorder who do not respond to previous treatments. Although its efficacy in reducing clinical symptomatology has been proven, few studies have analyzed its effects at the cognitive level. The aim of this systematic review was to describe the cognitive outcomes of functional neurosurgery in patients that went through capsulotomies or cingulotomies. PubMed, Medline, Scopus, PsycInfo, PsyArticles, and Web of Knowledge were searched for studies reporting cognitive outcomes in refractory obsessive–compulsive patients after capsulotomies and cingulotomies. The risk of bias was assessed with the Assessment Tool for Before-After (Pre-Post) Studies With No Control Group tool; 13 studies met inclusion criteria, including 205 refractory obsessive–compulsive disorder patients for both surgical procedures. Results showed a substantial number of studies that did report significant cognitive improvement after surgery, being this improvement specially related to memory and executive functions. The second-most frequent finding is the maintenance of cognitive performance (nor improvement or worsening). From a neuropsychological point of view, this outcome might be considered a success, given that it is accompanied by amelioration of obsessive–compulsive symptoms. Subtle cognitive adverse effects have also been reported. Neurosurgery procedures appear to be safe from a cognitive point of view. Methodological issues must be improved to draw clearer conclusions, but capsulotomies and cingulotomies constitute an effective alternative treatment for refractory obsessive–compulsive disorder patients.

**Supplementary Information:**

The online version contains supplementary material available at 10.1007/s10143-023-02037-w.

## Introduction

Obsessive–compulsive disorder (OCD) is a severe and disabling psychiatric disorder characterized by the presence of obsessions and/or compulsions [1, 2]. The international prevalence of this disorder is 1,1–1,8%, and of course, if not treated, it is chronic with oscillating symptoms [3]. In addition to clinical symptoms, cognitive impairments had been observed in this population in different domains, such as memory, executive functions, processing speed, and working memory [4–6]. First-line psychological and pharmacological treatments for patients with OCD are Cognitive-Behavioral Therapy (CBT) using Exposure with Response Prevention and pharmacotherapy with Selective Serotonin Reuptake Inhibitors (SSRI) [7–11]. If patients do not fully respond to one of these treatments or to the conjunction of both, augmentation strategies (e.g., adding antipsychotics) or switching to other drugs (e.g., clomipramine or other SSRIs) are recommended. However, even though many patients respond to these treatments, it is estimated that 40–60% of the patients remain refractory to pharmacological treatment [12] and that drop-out rates of CBT are between 9 and 16% [13]. For these cases with severe and highly refractory OCD, neurosurgery appears as a feasible therapeutic option [14, 15].

Neurosurgical procedures are used to improve specific symptoms caused by mental illnesses [16]. These are usually carried out by radiofrequency and radiosurgery but also by magnetic resonance-guided focused ultrasound (MRGFU) [17–19]. Specifically, capsulotomies and cingulotomies are considered the principal ablative procedures [19]. Capsulotomies refer to a technique in which a lesion is made in the anterior limb of the internal capsule (ALIC) from the frontal horn of the lateral ventricle along the head of the caudate nucleus [20]. By this lesion, frontothalamic connections are disrupted [21] and, specifically, the fibers of passage between the prefrontal cortex and subcortical nuclei, also comprising the dorsomedial thalamus [17]. On the other hand, cingulotomies refer to the procedures in which a lesion is made within the anterior cingulate cortex (ACC) [17], involving the cingulate gyrus and the white matter fibers of the cingulum bundle [21]. Even though the development of ablative surgery had been slowed in the past decades because of intense and public criticism [21], many studies have reported the efficacy of these procedures in reducing obsessive–compulsive symptomatology [e.g., 22, 23]. Moreover, considering the results of 278 patients who underwent anterior capsulotomies, Pepper, Zrinzo, and Hariz [24] reported clinical response in 73% of the patients and remission in 24% of patients, with a low rate of complications. In addition to capsulotomies, Brown et al. [25] also described improvement in OCD symptomatology after cingulotomy. Furthermore, neuroablation appears to have a lower complication rate than other procedures, such as deep brain stimulation (DBS) [26].

As observed, the assessment of the effectiveness of these procedures has been analyzed in terms of obsessive–compulsive symptom reduction, but results in terms of safety regarding the comparison of cognitive performance before and after surgery are scarce. Considering that the presence of different behavioral or cognitive impairments is frequent after brain damage [27], it is essential to describe whether the ablation of gray and white matter structures and whether the modification or interruption of brain tracts in controlled injuries does have a deleterious effect on a patient’s cognitive outcomes. In contrast, a recent systematic review and meta-analysis (i.e., Lai et al. [28]) about the clinical advantages and disadvantages of different neurosurgical interventions did not include or have omitted a detailed analysis of cognitive outcomes after surgery. This is an important issue, given that the analysis of the safety and the effectiveness of surgical procedures could be limited to the study of impairments at psychopathological and physiological levels (e.g., hemorrhages, infections) while ignoring the possibility of adverse cognitive effects. Derived conclusions could help to determine whether carrying out these interventions is convenient. In that case, empirical support would validate this technique, which in the most severe cases could help to improve the patient’s symptoms, and also their cognitive performance.

For this reason, the aim of the present study was to describe the cognitive outcomes of neurosurgery in patients that went through ablative functional neurosurgeries. To do so, a systematic review was conducted reporting the changes in neuropsychological performance found in studies of patients with refractory OCD who had undergone capsulotomies and cingulotomies. In this way, it is defined which cognitive functions are improved or impaired by neurosurgeries and which procedure seems to be safer when considering cognitive performance after functional neurosurgery in the treatment of refractory OCD.

## Materials and methods

### Literature search

**Fig. 1:**
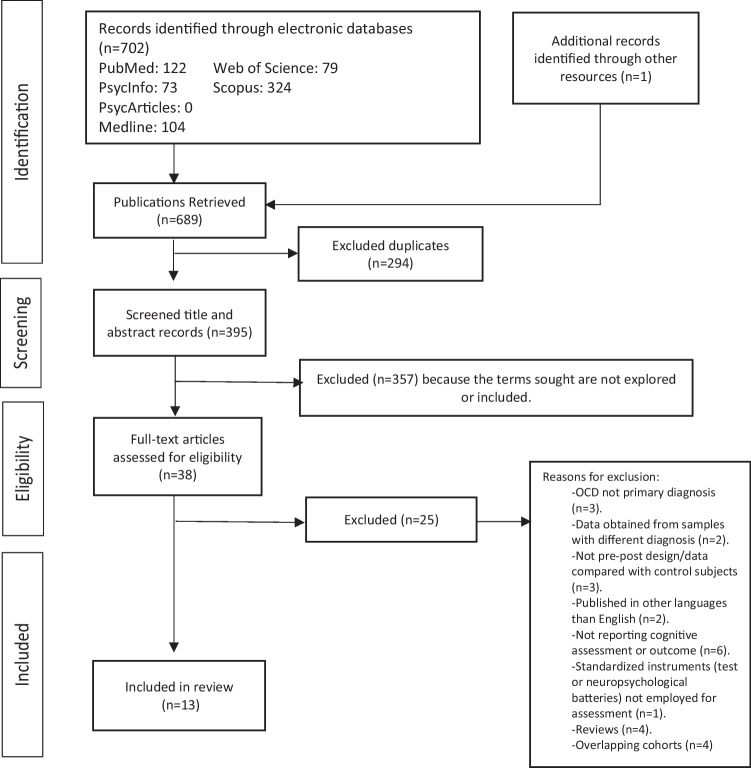
PRISMA-based flow diagram of the systematic review (Liberati et al. 2009; Moher et al. 2009)

The systematic review has been conducted following the Preferred Reporting Items for Systematic Reviews and Meta-Analyses (PRISMA) [29–31]. The PRISMA-based flow diagram (Fig. 1) illustrates the selection procedure. Specific search procedures and inclusion and exclusion criteria were established in a protocol and registered in PROSPERO (International Prospective Register of Systematic Reviews; registration number: CRD42023400038). This study was made in compliance with the Declaration of Helsinki.

### Search strategy

Publications about cognitive outcomes following neurosurgery (capsulotomies and cingulotomies) in patients with OCD were obtained from PubMed, Medline, Scopus, PsycInfo, PsyArticles, and Web of Knowledge databases and from backward searching citation chaining. In these six bibliographic databases, relevant published literature until January 2023 was searched using a combination of optimal search strategies including MESH terms “Obsessive–compulsive disorder,” “Cognitive Dysfunction,” “Neurosurgery,” and “Neurosurgical Procedures” if applied, or the terms “OCD,” “obsessive–compulsive disorder,” “obsessive–compulsive disorder,” “obsessive–compulsive,” “obsessive compulsive,” “cogn*,” “neuropsychol*,” “neurocogni*,” “neurosurgery,” “capsulotomy,” “cingulotomy,” “radiosurgery,” “radiofrequency,” “capsuloto*,” “cinguloto*,” and “psychosurgery” (Syntax, see Online resource 1). As mentioned, reference lists of included studies were consulted as an additional source for literature. At this point, no restrictions were included.

### Study selection and inclusion and exclusion criteria

Titles and abstracts of all identified were screened independently by two reviewers (G.L. and M.R). Duplicates of the reports were removed. In case of doubt, discrepancies in eligibility were resolved through discussion and consensus. After the selection of literature based on titles and abstracts, the full texts of the retained papers were assessed by reviewers to apply inclusion and exclusion criteria.

For the study selection, clear inclusion and exclusion criteria were followed and applied. Regarding study design, clinical trials (including randomized controlled trials, RCT) and observational studies (including cohort studies and case studies) with OCD patients that underwent neurosurgery (i.e., capsulotomies and cingulotomies) were included. Not only published articles but also data included in book chapters and conference abstracts were included. The included publications were all studies (a) which were published in English, (b) included patients with diagnosed OCD, and (c) 18–65 years aged. Finally, studies must report cognitive outcomes from patients with OCD who underwent capsulotomies and/or cingulotomies.

In addition, exclusion criteria included (systematic) reviews, letters, and animal studies, studies not reporting cognitive outcomes after neurosurgery or assessing those outcomes without standardized instruments (test or neuropsychological batteries), those including interventions other than neurosurgery, articles with designs different from pre-post design, those with samples which had a primary diagnosis other than OCD, and those with samples including patients with different diagnosis (without the possibility of extracting independently OCD data). Also, articles published in languages other than English, articles including patients aged < 18 or > 65 years were excluded, or those including patients with OCD symptoms attributable to medical cause (e.g., patients with the presence of OCD symptoms after streptococcal infection) [6].

### Qualitative assessment

The methodological quality and risk of bias assessment was performed using the National Heart, Lung, and Blood Institute Assessment Tool for Before-After (Pre-Post) Studies With No Control Group tool [32], according to Ma et al. [33] recommendations. Based on this resource, 12 questions about methodological issues were answered and studies were classified by the study overall quality (“poor,” “fair,” or “good”). No minimum quality score for inclusion was stated.

### Data collection

The data was extracted by one author (G.L.) using a standardized form and revised by two authors (J.A.P. and G.Lu). Patient data was compared among the studies to avoid the inclusion of the same sample in more than one report. In overlapping cohorts, the study with the longest follow-up was included. Where possible, details of the study design (sample), employed surgical technique, assessment (used cognitive tests or batteries), and other outcomes (subjective cognitive complaints or observations by relatives or physicians) were reported. Instruments and their scores were described and classified according to relevant OCD cognitive meta-analysis classification [6], reference guidelines in the neuropsychological field [34, 35], and validity studies [e.g., 36, 37]. In cases where the cognitive scores used were not explicitly available in the revised articles, the interpretation made by the authors was preserved, or no interpretation was included (reflected in Tables 2 and 3).

## Results

### Search results

In the initial search, 702 articles were identified, of which only 689 could be retrieved. After excluding 294 duplicates, 395 articles remained, of which titles and abstracts were screened. Then, 357 were excluded because the searched terms were not included or explored. Afterward, 38 publications remained, of which the full text was examined, which results in the exclusion of 25 other articles due to different reasons (see Fig. 1). The final sample comprised 13 articles. This final sample included 205 OCD refractory patients, of which 189 belong to studies that assessed cognitive outcomes after capsulotomies and only 19 to studies analyzing cognitive outcomes after cingulotomies. An overview of sample size, surgical procedures, and clinical features is included in Table 1. Cognitive measures, assessment times, and cognitive outcomes are reported in Table 2 and Table 3.

**Table 1:** Demographic and clinical characteristics of the samples of the studies revised

Authors	*n*	Operation details (RS, RF, TL-MRGFU)^a^	Settings (software, ablation equipment)^a^	Mean age symptoms initial occurrence (mean years, SD)^a^	Mean symptom/illness duration (mean years, SD)^a^	Mean age at surgery (mean years, SD)^a^	Follow-up (m)^a^	Mean Y-BOCS (Pre^a^)	Mean Y-BOCS (Post^a^)
Capsulotomies									
Fodstad et al. (1982)	2	RF	Air Ventriculography	Not reported	23	47	Before discharge, 1w, 1, 3, 6, 12, ≥ 12	Not reported	Not reported
Oliver et al. (2003)	15	RF – 75ºC	Brainlab, Radionics RF3	16.3 (9.6)	18.1 (5.6)	34.2 (8.2)	1, 2, 3, 6, 9, 12	29.67	19.12
Rück et al. (2008)	23	RF – 60º (*n* = 12) RS – 160 to 200 Gy (*n* = 9)	Gamma Knife (Elekta Instr.)	Not reported	21 (8)	41 (11)	12, 129	34.0 (3.4)	18.9 (12.0)
Csigó et al. (2010)	5	RF	Brainlab, Radionics	9 (6.74)	Not reported	32.2 (6.3)	1, 6, 12, 24	38.2 (1.78)	26.4 (5.12)
Jung et al. (2014)	4	TL-MRGFU – 51-56º	ExAblate 4000	Not reported	16.3 (5.7)	Not reported	1w, 1, 3, 6, 9	35.3 (1.9)	32 (3.3)
Zhan et al. (2014)	53	RF – 80º	Radionics RF3	Not reported	8.09 (2.99)	28.89 (9.16)	2w, 12, 36, 60	24.7 (4)	6.1 (4.8)
Batistuzzo et al. (2015)	17	RS-GK – 180 Gy (max)	Gamma Knife Model B (Elekta Instr.)	11.3 (5)	22.4 (12.8)	34.4 (10.6)	12, 24	32.9 (2.6)	21.4 (11.7)
Gong et al. (2018)	14	RF – 75º	SurgiPlan, Elekta neurostimulator	Not reported	12.6 (10)	31 (3.1)	1, 3, 6, 12	29.2 (5.37)	16.5 (4.78)
Kim et al. (2018)	11	TL-MRGFU – 51-56º	ExAblate 4000	Not reported	Not reported	Not reported	1w, 1, 3, 6, 12, 24	34.4 (2.3)	30.3 (4.3)
Peker et al. (2020)	21	RS-GK – 140–150 Gy	Gamma Knife 4C and Perfexion unit	Not reported	11.9 (5.23)	32.8 (7.61)	6, 12, 24, 36	35.7 (3.99)	29.9 (6.09)
Krámska et al. (2021)	12	RF-78–85°	SurgiPlan, 16-G, Diros Technology	18.17 (8.58)	21.42	39.7 (8.44)	6	31.67 (6.73)	16.58 (7.44)
Kassel et al. (2022)	9	RS-GK – 180 Gy (max)	Leksell Gamma Knife model U and C	Not reported	Not reported	Not reported	6, 12, 24, 60	33.6 (3.93)	22.68 (7.87)
Cingulotomies									
Fodstad et al. (1982)	2	RF	Air Ventriculography	Not reported	23	47	Before discharge, 1w, 1, 3, 6, 12, ≥ 12	Not reported	Not reported
Jung et al. (2006)	17	RF – 85º	Radionics RFG-3, Radionics	24.6 (9.8)	10.9 (5.2)	36.1 (9.4)	12, 24	35 (3.86)	22.4 (6.49)

*RS*, radiosurgery; *RF*, radiofrequency; *TL-MRGFU*, thermal lesioning by magnetic resonance-guided focused ultrasound; *SD*, standard deviation; *Y-BOCS*, yale-brown obsessive–compulsive scale; *NR*, not reported; *Gy*, grays; *w*, week; *RS-GK*, radiosurgery

^a^Data obtained from the full sample

**Table 2:** Studies reporting cognitive results after capsulotomies

Authors	Assessment times (months)	Cognitive measures (score)					Pre-score	Post-score	Cognitive outcome	Significance
Attention										
Kim et al. (2018)	B, 6, 12, 24	Digit span test (forward)^a^					10.2 (2.3)	10.1 (2.5)^b^	Not interpretable	No sign. (*p* > 0.05)
Visuospatial abilities										
Fodstad et al. (1982)	B, 6	FIT^a^ Kohs’ block test^a^ BVRT (number correct)^a^ BVRT (number error)^a^					Not reported^c^	Not reported^c^	Not interpretable	Not reported
Oliver et al. (2003)	B, 6, 12	TMT(A)					34.33	33.5^b^	No changes^d^	No sign. (*p* > 0.05)
Csigó et al. (2010)	B, 1, 6, 12, 24	TMT(A)					76.4 (45.08)	70.8 (72.42)^e^	No changes^d^	No sign. (*p* > 0.05)
Batistuzzo et al. (2015)	B, 24	TMT(A) BJLOT ROCFT (Copy)					54.56 (29.90) 24.08 (3.37) 31.82(3.79)	42.06 (19) 23.42 (3.12) 33.32 (8.19)	No changes except for TMT(A) – improvement	No sign. (*p* > 0.05) except TMT(A)
Peker et al. (2020)	B, 6	TMT(A)					28.5 (10.1)	24.4 (8.71)	No changes	No sign. (*p* > 0.05)
Krámska et al. (2021)	B, 6	ROCFT (copy) TMT(A) WAIS-III (block design subtest) WAIS-III (picture completion subtest)					32 (2.19) 40.5 (17.57) 32.57 (13.84) 18.09 (3.81)	32.17 (2.95) 38.25 (13.37) 38.2 (12.66) 19.25 (2.96)	No changes	No sign. (*p* > 0.05)
Memory (general)										
Fodstad et al. (1982)	B, 6	CMMT (object)^a^ CMMT (word-pair)^a^ CMMT (fig fact)^a^					Not reported^c^	Not reported^c^	Not interpretable	Not reported
Rück et al. (2008)	Not reported	Subjective complaints: subjective reports					Not applicable	Not applicable	Memory problems	Not applicable
Jung et al. (2014)	B, 6	RKMT (memory quotient)^a^					99.8 (4.9)	106 (10.9)	No changes	No sign. (*p* > 0.05)
Zhan et al. (2014)	Not reported	Subjective complaints: subjective reports					Not applicable	Not applicable	Impaired memory and confusion	Not applicable
Batistuzzo et al. (2015)	Not reported	Subjective complaints: subjective reports					Not applicable	Not applicable	Temporal and spatial disorientation	Not applicable
Kim et al. (2018)	B, 6, 12, 24	RKMT (memory quotient)^a^					94.1 (13.9)	103.3 (13.3)^b^	Improvement^d^	Sign. (*p* < 0.05)
Verbal memory										
Batistuzzo et al. (2015)	B, 24	HVLT-R (total recall) HVLT-R (delayed recall) HVLT-R (recognition hits) HVLT-R (learning) WMS-R (logical memory subtest, immediate recall) WMS-R (logical memory subtest, delayed recall)					24.08 (5.89) 7.67 (3.06) 10.92 (1.31) 4 (1.86) 22.18 (7.59) 17.88 (7.66)	23.50 (4.62) 7.67 (3.17) 11.33 (1.07) 2.92 (3.06) 24.18 (7.33) 21.47 (8.86)	No changes	No sign. (*p* > 0.05)
Gong et al. (2018)	B, 1, 3, 6, 12	WMS-R (logical memory subtest, immediate recall) WMS-R (logical memory subtest, delayed recall)					17.21 (5.62) 14.79 (6.10)	22.86 (5.52)^e^ 20.00 (6.00)^e^	Improvement^d^	Sign. (*p* < 0.05) except for delayed recall of WMS-R
Krámska et al. (2021)	B, 6	RAVLT (trials 1–5) RAVLT (long delay-free recall)					45.75 (12.7) 8.33 (3.52)	49.8 (12.12) 8.92 (3.55)	No changes	No sign. (*p* > 0.05)
Visual memory										
Fodstad et al. (1982)	B, 6	BVRT (number correct)^a^ BVRT (number error)^a^					Not reported^c^	Not reported^c^	Not interpretable	Not reported
Oliver et al. (2003)	B, 6, 12	WMS-R (recent visual memory) WMS-R (recall visual memory)					40.97 37.95	44.54^e^ 37.53^e^	No changes^d^	No sign. (p > 0.05)
Batistuzzo et al. (2015)	B, 24	ROCFT (delayed recall) BVMT-R (total recall) BVMT-R (delayed recall) BVMT-R (discrimination index) BVMT-R (learning)					9.82 (7.45) 17.82 (5.88) 7.27 (2.37) 4.45 (1.29) 4.36 (2.38)	14.5 (7.68) 21.45 (8.86) 8.36 (2.98) 4.91 (1.81) 3.64 (2.25)	No changes except ROCFT (delayed recall) – improvement	No sign. (*p* > 0.05) except ROCFT – delayed recall
Gong et al. (2018)	B, 1, 3, 6, 12	WMS-R (immediate visual reproduction) WMS-R (delayed visual reproduction)					9.79 (3.89) 8.57 (3.69)	13.36 (0.63)^e^ 12.21 (1.67)^e^	Improvement^d^	Sign. (*p* < 0.05)
Krámska et al. (2021)	B, 6	ROCFT (immediate recall) ROCFT (delayed recall)					15.86 (5.26) 16.05 (5.44)	20.04 (7.12) 20.29 (7.12)	Improvement	Sign. (*p* < 0.05)
Language										
Batistuzzo et al. (2015)	B, 24	BNT WASI (vocabulary subtest) WASI (similarities subtest)					53.36 (5.37) 46.47 (11.92) 32.31 (6.57)	54.42 (5.42) 53.53 (9.45) 34.82 (8.07)	No changes except WASI (vocabulary) – improvement	No sign. (*p* > 0.05) except WASI (vocabulary)
Gong et al. (2018)	B, 1, 3, 6, 12	WASI (similarities subtest)					17.57 (2.85)	17.35 (2.59)^e^	No changes^d^	No sign. (*p* > 0.05)
Krámska et al. (2021)	B, 6	WAIS-III (similarities subtest)					24.5 (5.14)	24.67 (4.48)	No changes	No sign. (*p* > 0.05)
Working memory										
Oliver et al. (2003)	B, 6, 12		TMT(B)		35.94			40.43^b^	No changes^d^	No sign. (*p* > 0.05)
Rück et al. (2008)^f^	B, 129		WAIS – R NI—digit span^a^		47 (8.6)			41 (10.3)	Not interpretable	Not reported
Csigó et al. (2010)	B, 1, 6, 12, 24		Corsi spatial working memory test TMT(B)		3.4 (0.74) 229.4 (137.89)			3.2 (1.03)^b^ 201.6 (100.33)^b^	No changes^d^	No sign. (*p* > 0.05)
Batistuzzo et al. (2015)	B, 24		TMT(B) TMT(B) (errors)		135.38 (76.6) 0.64 (1.03)			136.19 (79.3) 0.82 (0.87)	No changes	No sign. (*p* > 0.05)
Peker et al. (2020)	B, 6		TMT(B)		53.5 (14.4)			50.7 (12.4)	No changes	No sign. (*p* > 0.05)
Krámska et al. (2021)	B,6		TMT(B) WAIS-III (arithmetic subtest) WAIS-III (digit span subtest)		87.08 (40.54) 14.36 (3.64) 15.67 (4.44)			74.25 (43.62) 15 (3.59) 16.25 (4.27)	No changes	No sign. (*p* > 0.05)
Executive functions										
Rück et al. (2008)^f^	B, 129 Not reported		WCST Subjective complaints: EAD scale and subjective reports		41 (9.9) Not applicable			29 (13.9) Not applicable	Not interpretable Executive dysfunction, apathy, and disinhibition	Not reported Not applicable
Csigó et al. (2010)	B, 1, 6, 12, 24		CST-A (right concept)^a^ CST-B (perseverative errors)^a^ IGT (selection from disadvantageous desks)^a^ IGT (selection from advantageous desks)^a^ Stroop test (interference scale, time)^a^		3 (1.22) 3.6 (1.67) 57.4 (20.32) 42.6 (20.32) 39.6 (12.97)			5 (1)^e^ 2.8 (1.3)^e^ 50.6 (13.81)^b^ 49.2 (13.71)^b^ 35 (19.44)^e^	No changes except for CST-A (improvement^d^) and stroop test (interference scale, time) (not interpretable)	No sign. (*p* > 0.05) except for CST-A (right concept) and stroop test (interference scale, time)
Jung et al. (2014)	B, 6		Stroop test (numbers per second)^a^		0.87 (0.4)			0.91 (0.3)	No changes	No sign. (*p* > 0.05)
Zhan et al. (2014)	Not reported		Subjective complaints: subjective reports		Not applicable			Not applicable	Mild hypersexuality, apathy, initiative, and lack of interest-related problems	Not applicable
Batistuzzo et al. (2015)	B, 24 Not reported		VST (stroop 1 – time) VST (stroop 2 – time) VST (stroop 3 – time) VST (effect-time) WCST (64 cards) (hits) WCST (64 cards) (categories) WCST (64 cards) (set-loss) WCST (64 cards) (perseverative responses) WCST (64 cards) (perseverative errors) WCST (64 cards) (non-perseverative errors) ROCFT (planning score)^a^ TMT (effect–time) Subjective complaints		15.56 (3.09) 18.81 (5.26) 30.95 (7.75) 15.39 (6.13) 41.53 (8.64) 2.6 (1.18) 0.6 (0.63) 4.47 (9.99) 4.07 (8.96) 18.4 (8.54) 2.55 (1.44) 80.81 (56.90) Not applicable			15.82 (3.29) 18.66 (4.32) 28.94 (6.48) 13.12 (3.77) 45.63 (7.34) 3.13 (1.36) 0.19 (0.4) 3.56 (6.97) 3.19 (6.02) 15.31 (6.57) 2.91 (1.81) 94.13 (69.30) Not applicable	No changes except WCST (hits) and TMT (effect-time) – improvement Disinhibition	No sign. (*p* > 0.05) except WCST (hits) and TMT (effect-time) Not applicable
Gong et al. (2018)	B, 1, 3, 6, 12 Not reported		WCST (correct) WCST (errors) WCST (perseverative errors) WCST (non-perseverative errors) WCST (categories) Subjective complaints: subjective reports		24.93 (7.5) 23.07 (7.5) 16.86 (9.53) 6.21 (2.36) 3.86 (1.56) Not applicable			33.29 (5.7)^e^ 14.71 (5.7)^e^ 6.86 (5.86)^e^ 7.86 (1.96)^e^ 5.14 (0.95)^e^ Not applicable	Improvement^d^ except for WCST – (non-perseverative errors) – no changes^d^ Childish behavior, sexual disinhibition, lack of interest, decreased motivation, inappropriate laughter	Sign. (*p* < 0.05) except in WCST (non-perseverative errors) Not applicable
Kim et al. (2018)	B, 6, 12, 24		KCWST (mean reaction time for correct trials)^a^		1.25 (0.32)			1.30 (0.48)^b^	No changes^d^	No sign. (*p* > 0.05)
Peker et al. (2020)	B, 6		Stroop test (word score) Stroop test (color score) Stroop test (color-word score)		81.2 (8.83) 51.6 (7.91) 35.4 (4.71)			82.2 (7.72) 52.2 (6.73) 36.9 (8.11)	No changes	No sign. (*p* > 0.05)
Kassell et al. (2022)	B, 6, 12, 24–48, ≥ 60		WCST (128 cards) (perseverative errors) WCST (128 cards) (learning to learn) FrSBe (self-score) (item 13) FrSBe (family score) (item 13)		21.94 2.77 Not reported^c^ Not reported^c^			20.30^b^ 4.15^b^ Not reported^c^ Not reported^c^	Not interpretable No changes^d^	Not reported No sign. (*p* > 0.05)
Verbal fluency										
Csigó et al. (2010)	B, 1, 6, 12, 24		Verbal fluency test (number of words)^a^ Verbal fluency test (perseverative errors)^a^ Category fluency test (intrusion errors)^a^		32.4 (3.78) 1 (1) 0.6 (0.89)			46.4 (10.73)^e^ 1.8 (4.02)^e^ 2.6 (2.51)^e^	Improvement^d^ in VFT – (number of words) No changes^d^ in VFT – (pers.errors) Worsening^d^ in CFT-(intrusion errors)	Sign. (*p* < 0.05) except in VFT (pers. errors)
Jung et al. (2014)	B, 6		COWAT (phonemic portion) COWAT (semantic portion)		38 (16.8) 19.3 (6.8)			37 (16.3) 20.3 (3.3)	No changes	No sign. (*p* > 0.05)
Kim et al. (2018)	B, 6, 12, 24		COWAT (phonemic) COWAT (semantic)		35.5 (15) 19.5 (6.4)			38.2 (20.6)^b^ 18.3 (5.8)^b^	No changes^d^ No changes^d^	No sign. (*p* > 0.05)
Krámska et al. (2021)	B, 6		COWAT (phonemic fluency)		37.58 (11.79)			36.75 (9.95)	No changes	No sign. (*p* > 0.05)
Information processing speed										
Krámska et al. (2021)	B, 6		Digit symbol from WAIS-III		47 (13.08)			53.25 (15.05)	No changes	No sign. (*p* > 0.05)
Intelligence										
Fodstad et al. (1982)	B, 6		SRB-Test – synonyms^a^ SRB-Test – reasoning^a^		Not reported^c^			Not reported^c^	Not interpretable	Not reported
Oliver et al. (2003)	B, 6, 12		Koh’s cubes WAIS (IQ)		44.83 98.88			41^e^ 93.77^e^	No changes^d^	No sign. (*p* > 0.05)
Csigó et al. (2010)	B, 1, 6, 12, 24		MAWI (Weshcler intelligence quotient)^a^		91.8 (10.94)			107.8 (7.04)^e^	Improvement^d^	Sign. (*p* < 0.05)
Jung et al. (2014)	B, 6		K-WAIS		97.8 (21.7)			101 (23.8)	No changes	No sign. (*p* > 0.05)
Zhan et al. (2014)	B, 2w, 12, 36, 60		WAIS		Not reported			Not reported	No changes^d^	No sign. (*p* > 0.05)
Batistuzzo et al. (2015)	B, 24		WASI (verbal IQ) WASI (performance IQ) WASI (total IQ) WASI (block design subtest) WASI (matrix reasoning subtest)		91.38 (16.76) 89 (9.93) 90.74 (13.83) 29.31 (13.33) 20.41 (7.45)			97.41 (15.93) 93.25 (11.48) 94.13 (13.74) 31.38 (4.25) 21.82 (10.84)	Improvement for WASI (performance IQ, total IQ), and block Design subtest No changes for WASI (verbal IQ) and matrix reasoning subtest	Sign. (*p* < 0.05) except for WASI verbal IQ and matrix reasoning
Gong et al. (2018)	B, 1, 3, 6, 12		WASI (block design subtest)		31.50 (10.76)			40.36 (9.57)^e^	Improvement^d^	Sign. (*p* < 0.05)
Kim et al. (2018)	B, 6, 12, 24		K-WAIS^a^		90.0 (19.3)			93.5 (19.7)^b^	No changes^d^	No sign. (*p* > 0.05)
Krámska et al. (2021)	B, 6		WAIS-III (full-scale intelligence quotient)		101.82 (11.17)			102.83 (11.13)	No changes	No sign. (*p* > 0.05)
Motor functioning										
Fodstad et al. (1982)	B, 6			Zeal test^a^		Not reported^c^		Not reported^c^	Not interpretable	Not reported
Batistuzzo et al. (2015)	B, 24			GPT – dominant hand – time GPT – dominant hand – errors GPT – non-dominant hand – time GPT – non-dominant hand—errors HFTT – dominant hand HFTT – non-dominant hand HD – dominant hand HD – non-dominant hand		87.39 (17.25) 0.45 (0.52) 95.24 (19.5) 0.55 (0.82) 47.79 (9.48) 43.66 (7.09) 28.85 (8) 25.72 (8.39)		79.27 (18.89) 0.36 (0.67) 97.91 (32.05) 0.55 (0.69) 48.06 (9.06) 44.58 (6.17) 28.8 (8.19) 28.88 (8.42)	No changes except for GPT (dominant hand – time), and HD (non-dominant hand scores) – improvement	No sign. (*p* > 0.05) except GPT (dominant hand – time), and HD (non-dominant hand scores)
Overall cognitive functioning										
Zhan et al. (2014)	B, 2w, 12, 36, 60			MMSE		Not reported		Not reported	No changes^d^	No sign. (*p* > 0.05)
Batistuzzo et al. (2015)	B, 24			MMSE		91.94 (6.23)		92.69 (7.20)	No changes	No sign. (*p* > 0.05)
Peker et al. (2020)	B, 6			ACER (total score)		82.4 (7.2)		84.8 (8.6)	No changes	No sign. (*p* > 0.05)

*B*, baseline; *FIT*, figure identification test; *BVRT*, Benton’s visual retention test; *TMT*, trail making test; *BJLOT*, Benton judgement of line orientation test; *ROCFT*, Rey-Osterrieth complex figure test; *WAIS-III*, Weschler adult intelligence scale 3rd revision; *CMMT*, Cronholm-Molander memory test; *RKMT*, Rey-Kim memory test; *HVLT-R*, Hopkins verbal learning test-revised; *WMS-R*, Weschler memory scale-revised; *RAVLT*, Rey auditory verbal learning test; *BVMT-R*, brief visuospatial memory test-revised; *BNT*, Boston naming test; *WASI*, Weschler abbreviated scale of intelligence; *WAIS-R NI*, Weschler adult intelligence scale-revised as a neuropsychological instrument; *WCST*, Wisconsin card sorting test; *EAD*, execution, apathy and disinhibition scale; *CST*, California sorting test; *IGT*, Iowa gambling test; *VST*, Victoria stroop test; *KCWST*, Korean colour word stroop test; *FrSBe*, frontal systems behavior scale; *COWAT*, controlled association word association test; *WAIS*, Weschler adult intelligence scale; *MAWI*, Hungarian version of the Weschler intelligence test; *K-WAIS*, Weschler adult intelligence scale – Korean version; *GPT*, grooved pegboard test; *HFTT*, Halstead finger tapping test; *HD*, hand dynamometer; *MMSE*, mini mental state examination; *ACER*, Addenbrooke cognitive examination revised

^a^As the authors reported in the original article

^b^Post-score at the first post-assessment date

^c^Not results of the group; individual data is reported

^d^Considering all post-assessments reported in the article

^e^Post-score at the last post-assessment date

^f^Results of a subsample, *n* = 7

**Table 3:** Studies reporting cognitive results after cingulotomies

Authors	Assessment times (months)	Cognitive measures (score)	Pre-score	Post-score	Cognitive outcome	Significance
Visuospatial abilities						
Fodstad et al. (1982)	B, 6	FIT^a^ Kohs’ block test^a^ BVRT (number correct)^a^ BVRT (number error)^a^	Not reported^b^	Not reported^b^	Not interpretable	Not reported
Jung et al. (2006)	B, 12	ROCFT (copy)	Not reported^c^	Not reported^c^	No changes	No sign. (*p* > 0.05)
Memory (general)						
Fodstad et al. (1982)	B, 6	CMMT (object)^a^ CMMT (word-pair)^a^ CMMT (fig fact)^a^	Not reported^b^	Not reported^b^	Not interpretable	Not reported
Jung et al. (2006)	Not reported	Subjective complaints: subjective reports	Not applicable	Not applicable	Immediate memory dysfunction	Not applicable
Verbal memory						
Jung et al. (2006)	B, 12	HVLT (total number of words recalled during the three trials) HVLT (delayed recall) HVLT (learning) HVLT (percentage retained) HVLT (HVLT) (recognition)	Not reported^c^	Not reported^c^	No changes	No sign. (*p* > 0.05)
Visual memory						
Fodstad et al. (1982)	B, 6	BVRT (number correct)^a^ BVRT (number error)^a^	Not reported^b^	Not reported^b^	Not interpretable	Not reported
Jung et al. (2006)	B, 12	ROCFT (immediate recall) ROCFT (delayed recall)	Not reported^c^	Not reported^c^	No changes	No sign. (*p* > 0.05)
Executive functions						
Jung et al. (2006)	B, 12	WCST (total number of correct responses) WCST (total number of errors) WCST (perseverative responses) WCST (perseverative errors) WCST (non-perseverative errors)	Not reported^c^	Not reported^c^	Improvement except in WCST (total n. of correct responses) – no changes	Sign. (*p* < 0.05) except in WCST (total n. of correct responses)
Verbal fluency						
Jung et al. (2006)	B, 12	COWAT (total number of words in letter version) COWAT (total number of words in category version)	Not reported^c^	Not reported^c^	No changes	No sign. (*p* > 0.05)
Intelligence						
Fodstad et al. (1982)	B, 6	SRB-test – synonyms^a^ SRB-test – reasoning^a^	Not reported^b^	Not reported^b^	Not interpretable	Not reported
Jung et al. (2006)	B, 12	K-WAIS (full scale – IQ)^a^ K-WAIS (verbal – IQ)^a^ K-WAIS (performance– IQ)^a^	Not reported^c^	Not reported^c^	No changes	No sign. (*p* > 0.05)
Motor functioning						
Fodstad et al. (1982)	B, 6	Zeal test^a^	Not reported^b^	Not reported^b^	Not interpretable	Not reported

*B*, baseline; *FIT*, figure identification test; *BVRT*, Benton’s visual retention test; *ROCFT*, Rey-Osterrieth complex figure test; *CMMT*, Cronholm-Molander memory test; *HVLT*, Hopkins verbal learning test; *WCST*, Wisconsin card sorting test; *COWAT*, controlled association word association test; *K-WAIS*, Weschler adult intelligence scale – Korean version

^a^As the authors reported in the original article

^b^Not results of the group; individual data is reported

^c^Numerical data is not reported; data in graphics

The set of 13 revised manuscripts showed that up to 40 neuropsychological tests and batteries have been used to assess cognitive outcomes in patients with refractory OCD who had undergone capsulotomies and cingulotomies. In order to analyze the reported information, the following cognitive domains were distinguished (see Tables 2 and 3): attention, visuospatial abilities, memory (including verbal and visual memory), language, working memory, executive functions, verbal fluency, information processing speed, intelligence, motor functioning, and overall cognitive functioning. This was made according to the reference guidelines reported in the “Materials and method” section. Overall, 7 (54%) studies reported significant changes in neuropsychological scores after surgery (all of them reporting cognitive amelioration, and only one reporting cognitive worsening for one score analyzed), and 6 (46%) found no significant changes. The presented results are differentiated according to the target of the lesion performed (capsulotomies or cingulotomies). Finally, the results of the quality assessment are reported.

### Cognitive outcomes depending on the anatomical target

#### Capsulotomies

As observed in Table 2, capsulotomies are procedures in which the cognitive outcomes of OCD patients after surgery have been examined, showing significant changes in several domains.

First, there have been improvements consistently reported in the domains of memory and executive functions. Regarding memory, improvement has been found globally (memory quotient of the Rey-Kim memory test) [38] and in both verbal [39] and visual [39–41] modalities, the latter being more prominent. It should be noted that improvements occurred in tests that included both immediate and delayed recall scores [39–41], being the improvement registered by the Rey-Osterrieth complex figure test (ROCFT) the most frequent one (immediate recall [41], delayed Recall [40, 41]). Additionally, it should be noted that improvements in verbal memory were reported (scores of the immediate and delayed recall of the logical memory subtest of the Weschler memory scale-revised (WMS-R) [39]). Regarding executive functions, improvements are found specifically in California sorting test-A (right concept) [42], Wisconsin card sorting test (WCST) (hits, errors, perseverative errors, and categories scores) [39, 40], TMT effect-time score [40], and stroop (interference time score) [42]. However, the interpretation of this last score is not straightforward (since it differs from the standardized form and the authors did not provide additional information).

Secondly, scores in motor functioning, visuospatial abilities, language, and verbal fluency domains have shown significant changes. These scores show improvements in the grooved pegboard test (dominant hand-time), hand dynamometer (non-dominant hand), trail making test A (TMT-A), vocabulary subtest from Weschler abbreviated scale of intelligence (WASI) [40], and verbal fluency test (number of words) [42]. In addition, there have also been reported improvements in general intelligence [total intelligence quotient (IQ) of WASI, IQ of Hungarian version of the Weschler intelligence test (MAWI) [40, 42] and specific components of intelligence (performance IQ, block design subtest of WASI) [39, 40].

Thirdly, the only worsening recorded to date after capsulotomies has been found in verbal fluency tasks (intrusion errors in the category fluency test) [42].

Fourthly, no significant changes were found in neuropsychological performance in attention, working memory, information processing speed, and overall cognitive functioning (see Table 2).

Fifthly, when studying subjective complaints, or behavioral observations, 7 studies reported no cognitive adverse effects [38, 41–46], while other 4 reported the presence of executive dysfunction, apathy, disinhibition symptoms, disorientation (temporal and spatial), childish behavior, lack of interest, decreased motivation, inappropriate laughter and impaired memory, and confusion [39, 40, 47, 48] after surgery. Only one study used a scale to register subjective complaints (EAD scale [47]), while the others used clinical observation or spontaneous reports of the patients or caregivers [38, 45], but in most cases, the method used to register the presence of subjective effects was not stated [39–41, 43, 44, 46].

Finally, regarding assessment times, of the 12 studies that examined changes in neuropsychological performance, most conducted the post-surgical assessments within the first year after surgery [39, 41–46, 49,48], extending the post-assessments in some cases to two years [38, 40, 42] or more [47–49]. Of those that performed post-evaluation during the first year after surgery, the majority were radiofrequency studies [39, 41, 43, 46], followed by radiosurgeries [47] and thermal lesioning [44]. The remaining studies performed evaluations in a period equal to or longer than two years and included radiofrequency [42, 47, 48], radiosurgery [40, 47, 49], and thermal lesioning [38] procedures. Considering the assessment times of those 11 studies analyzing subjective complaints, two reported subjective cognitive adverse effects immediately after surgery [47, 48], another one at 8 months [39], and two at 12 months after surgery [39, 47]. Three studies reported the assessment times of subjective complaints: Jung et al. [44] reported that this assessment was made at 6 months, Kim et al. [38] in every follow-up visit (i.e., 1 week, 1 month, 3 months, 6 months, 12 months, and 24 months after surgery) and Peker et al. [45] also in every appointment (i.e., 6 months and annually until fifth year after surgery). However, the eight remaining articles did not indicate an exact indication of which time they carried out the assessment of subjective complaints [39–43, 46–48].

#### Cingulotomies

As seen in Table 3, to date, only one study reported significant improvements, found in the domain of executive functions, as reflected in the scores of WCST (total number of errors, perseverative responses, perseverative errors, and non-perseverative errors) [50]. In addition, it should be noted that no pre-post cognitive neuropsychological changes had been studied in attention, language, information processing speed, and overall cognitive functioning domains. Regarding subjective complaints, one study reported no worsening [43], and one study described the presence of subjective immediate memory dysfunction complaints that were not found by means of standardized neuropsychological assessment [50].

Considering the cognitive assessment times, the two studies included for cingulotomies performed neuropsychological assessments within the first year after surgery, and both followed radiofrequency procedures [43, 50]. One of them reported subjective complaints that were registered 2–3 months after surgery [50].

### Quality assessment

In brief, qualitative assessment rated five studies as “poor” (38%), four as “fair” (31%), and four as “good” (31%) (see Online resource 2) [32].

## Discussion

In the present review, studies analyzing cognitive outcomes in patients with OCD after ablative neurosurgery (capsulotomies and cingulotomies) have been revised. The main result was that a substantial number of studies report cognitive amelioration in neuropsychological performance after surgery. This improvement appears to be multi-domain, although it is more frequently reported for memory and executive functions for both types of surgeries. In addition, many studies have shown no cognitive changes (nor improvement nor worsening), what can be considered a successful outcome, suggesting that surgery is a safe and potentially curative procedure for many patients without showing adverse effects. It appears that increases in neuropsychological performance may be quite extensive in capsulotomies, but subtle deleterious effects have also been reported. No worsening in neuropsychological functioning has been reported for cingulotomies, although some patients exposed subjective complaints that should be studied in more detail. Overall, neurosurgery has shown the presence of improvements that are clearly superior to the possible worsening detected.

In capsulotomies, a heterogeneous pattern of cognitive improvements has been reported, although the body of the results is consistently related to memory and executive functions. Improvements have also been found in domains such as motor functioning, visuospatial abilities, language, and verbal fluency. In this regard, the pathological activity of the hyperactive orbito-fronto-striato-thalamo-cortical circuitry that is associated with OCD [51–53] appears to be modified by the intervention in fiber tracts between orbitofrontal cortex (OFC) and thalamus by targeting the ventral fibers of ALIC [54]. An interruption of this circuit, and the amelioration of obsessive–compulsive symptoms, may lead to secondary restoration of the activity of the networks that relate to frontal executive abilities [55]. Indeed, executive impairments appear to promote the exacerbation of OCD symptoms [56]. So, an improvement in neuropsychological performance might relate to a redistribution of cognitive resources that previously could be involved in clinical symptoms. This last idea is also similar to the capacity-reduction hypothesis in disorders such as depression. People with depression present a cognitive capacity reduction that is exhibited in effortful tasks (including cognitive tasks) because available cognitive resources are focused on negative self-content and fewer attentional resources are displayed. Certainly, interference in effortful processing has been associated with psychopathology in general [57, 58]. Therefore, effective treatment of clinical symptoms (also in OCD) may allow for optimum distribution of the available attentional capacity and, in consequence, better neuropsychological performance. Along with the mentioned improvement, worsening has also been found for one executive score in only one study (i.e., intrusion errors of category fluency test) [42], as well as executive and memory subjective cognitive complaints in 4 studies [39, 40, 47, 48]. According to the literature, if worsening occurs, it might be related to the localization of the target area within the ALIC, where more dorsally located lesions might be related to impairments in different cognitive domains (i.e., executive functions and working memory) [59]. However, ventral lesions of this structure seem to involve the longitudinal fiber tracts that connect anterior cingulate and orbitofrontal projections to the striatum, thalamus, and brainstem, reducing the adverse effects without compromising the therapeutic effects [28]. In this way, results with an almost complete absence of cognitive worsening could denote preservation of the ALIC fibers that could cause cognitive impairments when lesioned [59].

In cingulotomies, the explanation of cognitive changes associated with neurosurgery is challenging given that the available literature is modest and the cognitive domains studied had been a few. On the one hand, the main finding is a general cognitive improvement, especially in executive functions, while no significant changes have been found in other cognitive functions. Kim et al. [60] stated that improvements in WCST scores (similar to those of Jung et al. [50]) reflect improvements in the capacity to flexibly change behavior as the environment changes (cognitive flexibility) and also to monitor and adapt behavior (cognitive control) [50, 60–62]. This last domain is specially related to the main target of cingulotomies, the dorsal anterior cingulate cortex (dACC), which contributes to response monitoring, response inhibition, and goal selection [59]. According to the expected value of control theory, the dACC would exhibit an abnormal function in OCD (i.e., would be pathologically active) and would lead to behaviors related to OCD symptoms. Particularly, the dACC would present problems in the specification of the identity and/or intensity of signals (called cognitive control signals) produced to alert of possible dangers. Even if the threat is not real or does not longer exist, the control signals persist, leading to the emergence of repetitive behaviors that are not adaptative to respond to stimuli interpreted as highly threating [63, 64]. In this regard, it is possible that cingulotomies would reduce the activity of the dACC and normalize its function. This would lead to the allocation of cognitive resources that were previously involved in generating behaviors in response to perceived threats [59] to the tasks of interest. On the other hand, and considering subjective cognitive complaints, only one of the two studies on cingulotomies stated cognitive adverse effects related to memory [50]. In this view, the cingulate gyrus has been associated with memory function by means of its connections with the retrosplenial cortex, hippocampal, and parahippocampal regions [65, 66], so the extension of the lesion in cingulotomies to proximal regions could, in turn, have an impact on memory mechanisms. However, in the absence of a more detailed study of cognitive control and the role and connections of the cingulate cortex, determining how cingulotomies impact executive functions and memory in OCD is still a complex purpose.

From a methodological point of view, the revised studies show a heterogeneity that makes it difficult to implement a direct comparison among them. Firstly, the differences in study design have possibly conditioned the reporting of results (i.e., case studies [43]). Secondly, the inclusion and exclusion criteria may have influenced the possibility of detecting cognitive changes due to a ceiling effect. Thus, Jung et al. [44] and Kim et al. [38] excluded all patients that had an MMSE score lower or equal to 24, Zhan et al. [48] did not include patients that had cognitive deficits, and Peker et al. [45] excluded those patients with cognition in the “low range.” Given this, the exclusion of participants with the worst cognitive performance might cause, in an artifactual way, difficulties in detecting cognitive improvement that could occur since patients whose performance would likely improve are eliminated. In those studies in which only patients with normal cognitive performance are evaluated, the possibilities of detecting changes are clearly limited by the small margin for improvement that exists. In fact, in three of these four studies, no significant changes were detected in neuropsychological pre-post comparisons. Thirdly, three different neurosurgery techniques have been detected in the revised literature (radiosurgery, radiofrequency, and thermal lesioning by focused ultrasound) (e.g., [44, 46, 49]), which could influence the results according to their mechanisms of action. Indeed, the time course of clinical response to radiosurgery (onset of symptom improvement has been reported from 3 months to 3 years; [14]) is different from other procedures such as radiofrequency, where the gradual nature of the radiation effects is characteristic [19]. This raises the idea that the timing of neuropsychological assessments may be conditioned by the time course of the effects of such techniques. In the case of Peker’s et al. [45] radiosurgery study, this assumption is relevant given that the follow-up was made 6 months after surgery which could be a premature time for the detection of the changes. However, for all the other studies, the times selected for the cognitive assessment were adequate according to the type of technique, so the cognitive changes that might have taken place have probably been captured. By extension, this issue ties in with the possibility of detecting cognitive changes (improvement or worsening) within the first year after surgery. Along with this idea, improvement of executive functions and memory may evolve in the second year after surgery [38, 42]. Therefore, for studies that did not show changes in cognitive performance, and with the neuropsychological follow-up taking place in the first six or twelve months after surgery (e.g., [44–46], it cannot be ruled out that a longer period of time was necessary for the improvement in cognitive performance to take place and, consequently, be detected. Fourthly, some studies included tests that were difficult to access (e.g., [43]), or have used versions adapted to demographic characteristics of the sample (but with the not available explanation of these adaptations), so direct interpretation and comparison of the results was not possible (e.g., K-WAIS [44]; Korean colour word stroop test [38]; category fluency test [42]). In other cases, no scores were indicated or relevant data were missing (e.g., numerical data or *p*-value), making it difficult to interpret the results [38, 43, 44, 47–50]. Moreover, the realization of an adequate neuropsychological assessment along with standardized instruments for the detection of cognitive impairments is a fundamental issue in the neurosurgical context since patients with different cognitive profiles are linked to distinct clinical profiles [67] that appear to benefit differently from capsulotomies [68]. In this line, the differences between the worsening detected by neuropsychological performance and by subjective cognitive adverse reporting for both surgeries must be considered, and it may be appropriate to suggest that some impairments may not have been recorded in neuropsychological assessment. This fact is relevant since it was not possible to assess how cognitive impairments have impacted the patient’s daily functioning through the tests and batteries employed in the revised studies. It can also be added that those patients referring to subjective complaints could be more aware of executive and memory impairments, considering their notable interference with daily life [e.g., 69]. Thus, it would be necessary to build a protocol in which subjective complaints are registered in a systematic way. Lastly, the quality of the studies that make up this review should be afforded. The number of studies that have been assessed to be “poor” or “fair” quality is noteworthy (69%), exceeding the number of those considered to have “good” quality (31%). In this vein, there is a clear need to improve the methodological quality of studies with standardized procedures according to the established consensus of the neurosurgery community [e.g., 70]. However, when the studies rated as “good” are examined, it is possible to observe a very similar pattern of results showing consistent improvements in executive functions and memory [39–41]. Indeed, no worsening scores are found in this group of studies. Therefore, although methodological difficulties are reported in most of the revised manuscripts, and results should be addressed with caution, derived conclusions converge among studies.

## Conclusions

Among the revised literature, ablative functional neurosurgery appears to be a safe procedure from the cognitive or neuropsychological point of view. Memory and executive functions appear to be the domains in which the most marked improvement has been found. In addition, a great proportion of studies did not show significant cognitive changes after neurosurgery which might also be considered a successful outcome after surgery. In conclusion, neurosurgery procedures appear to be an alternative treatment option for refractory OCD patients, but methodological issues related to neuropsychological or cognitive assessment should be improved to further detail the changes and cognitive outcomes and to draw clearer conclusions about the efficacy of the procedures.

### Supplementary Information

Below is the link to the electronic supplementary material.

Supplementary file1 (DOCX 31 KB)

## Data Availability

Not applicable.
