# Supplementary material for: Cognitive outcomes following functional neurosurgery in refractory OCD patients: a systematic review
Source: Neurosurg Rev. 2023 Jun 23;46(1):145. doi: 10.1007/s10143-023-02037-w (PMC10289910; doi:10.1007/s10143-023-02037-w)
Supplement: Supplementary file 1 — Supplementary file1 (DOCX 31 KB) [file 10143_2023_2037_MOESM1_ESM.docx]

Supplementary Materials

1. Search Strategy.

Literature search was made in January 2023, and results included all studies found up to that date.

1.1. PubMed Search Strategy.

| Search order | Terms | Number of results |
| --- | --- | --- |
| 1 | Obsessive-Compulsive Disorder [MeSH Terms] | 16,461 |
| 2 | obsessive-compulsive disorder [Title/Abstract] | 15,288 |
| 3 | obsessive compulsive disorder [Title/Abstract] | 15,288 |
| 4 | obsessive-compulsive [Title/Abstract] | 20,761 |
| 5 | obsessive compulsive [Title/Abstract] | 20,761 |
| 6 | OCD [Title/Abstract] | 11,609 |
| 7 | #1 OR #2 OR #3 OR #4 OR #5 OR #6 | 26,429 |
| 8 | Cognitive Dysfunction [MeSH Terms] | 33,755 |
| 9 | neurocogniti* [Title/Abstract] | 29,241 |
| 10 | cogn* [Title/Abstract] | 538,234 |
| 11 | neuropsychol* [Title/Abstract] | 63,417 |
| 12 | #8 OR #9 OR #10 OR #11 | 576,780 |
| 13 | Neurosurgery [MeSH Terms] | 222,693 |
| 14 | Neurosurgical Procedures [MeSH Terms] | 211,422 |
| 15 | neurosurgery [Title/Abstract] | 30,275 |
| 16 | radiosurgery [Title/Abstract] | 15,113 |
| 17 | radiofrequency [Title/Abstract] | 44,565 |
| 18 | capsuloto* [Title/Abstract] | 3,604 |
| 19 | cinguloto* [Title/Abstract] | 213 |
| 20 | psychosurgery [Title/Abstract] | 2,304 |
| 21 | #13 OR#14 OR #15 OR #16 OR #17 OR #18 OR #19 OR #20 | 288,126 |
| 22 | #7 AND #12 AND #21 | 122 |

1.2. PsycInfo Search Strategy

AB(obsessive-compulsive disorder OR obsessive compulsive disorder OR OCD) AND AB(neurocogniti* OR cogn* OR neuropsychol*) AND AB(neurosurgery OR capsuloto* OR cinguloto* OR radiosurgery OR radiofrequency OR psychosurgery)

Total founded studies: 73.

1.3. PsycArticles Search Strategy

AB (obsessive-compulsive disorder OR obsessive compulsive disorder OR OCD) AND AB(neurocogniti* OR cogn* OR neuropsychol*) AND AB(neurosurgery OR capsuloto* OR cinguloto* OR radiosurgery OR radiofrequency OR psychosurgery)

Total founded studies: 0.

1.4. MEDLINE Search Strategy

AB (obsessive-compulsive disorder OR obsessive compulsive disorder OR OCD) AND AB (neurocogniti* OR cogn* OR neuropsychol*) AND AB (surgery OR psychosurgery OR radiosurgery OR radiofrequency OR capsuloto* OR cinguloto*)

Total founded studies: 104.

1.5. Scopus Search Strategy

TITLE-ABS-KEY({obsessive-compulsive disorder} OR {obsessive compulsive disorder} OR {obsessive-compulsive} OR {obsessive compulsive} OR ocd) AND TITLE-ABS-KEY(neurocogni* OR cogn* OR neuropsychol*) AND TITLE-ABS-KEY(neurosurgery OR radiosurgery OR radiofrequency OR capsuloto* OR cinguloto* OR psychosurgery).

Total founded studies: 324.

1.6. Web of Science Search Strategy

| Search order | Terms | Number of results |
| --- | --- | --- |
| 1 | AB=(obsessive-compulsive disorder) | 16,489 |
| 2 | AB=(obsessive compulsive disorder) | 16,555 |
| 3 | AB=(obsessive-compulsive) | 18,085 |
| 4 | AB=(obsessive compulsive) | 18,230 |
| 5 | AB=(OCD) | 11,965 |
| 6 | #1 OR #2 OR #3 OR #4 OR #5 | 20,697 |
| 7 | AB=(neurocogniti*) | 25,530 |
| 8 | AB=(cogn*) | 690,366 |
| 9 | AB=(neuropsychol*) | 55,651 |
| 10 | #7 OR #8 OR #9 | 723,395 |
| 11 | AB=(neurosurgery) | 15,406 |
| 12 | AB=(capsulotomy) | 2,368 |
| 13 | AB=(cingulotomy) | 146 |
| 14 | AB=(radiosurgery) | 11,742 |
| 15 | AB=(radiofrequency) | 36,044 |
| 16 | AB=(capsuloto*) | 2,460 |
| 17 | AB=(cinguloto*) | 152 |
| 18 | AB=(psychosurgery) | 196 |
| 19 | #11 OR #12 OR #13 OR #14 OR #15 OR #16 OR #17 OR #18 | 65,282 |
| 20 | #6 AND #10 AND #20 | 79 |

2. Qualitative assessment following Assesment Tool for Before-After (Pre-Post) Studies With No Control Group (NIH, 2021)

| Questions | Fodstad et al. (1982) | Oliver et al.  (2003) | Rück et al. (2008) | Csigó et al. (2010) | Jung et al. (2014) | Zhan et al.,  (2014) | Batistuzzo et al.  (2015) |
| --- | --- | --- | --- | --- | --- | --- | --- |
| 1. Was the study question or objective clearly stated? | No | Yes | Yes | Yes | Yes | Yes | Yes |
| 2. Were eligibility/selection criteria for the study population prespecified and clearly described? | No | Yes | Yes | Yes | Yes | C.D. | Yes |
| 3. Were the participants in the study representative of those who would be eligible for the test/service/intervention in the general or clinical population of interest? | No | C.D. | Yes | Yes | Yes | No | Yes |
| 4. Were all eligible participants that met the prespecified entry criteria enrolled? | Yes | No | No | Yes | Yes | Yes | No |
| 5. Was the sample size sufficiently large to provide confidence in the findings? | No | Yes | Yes | Yes | No | Yes | Yes |
| 6. Was the test/service/intervention clearly described and delivered consistently across the study population? | Yes | Yes | No | No | Yes | Yes | Yes |
| 7. Were the outcome measures prespecified, clearly defined, valid, reliable, and assessed consistently across all study participants? | Yes | Yes | No | No | Yes | Yes | Yes |
| 8. Were the people assessing the outcomes blinded to the participants' exposures/interventions? | No | No | No | No | No | No | Yes |
| 9. Was the loss to follow-up after baseline 20% or less? Were those lost to follow-up accounted for in the analysis? | Yes | Yes | Yes | Yes | Yes | Yes | Yes |
| 10. Did the statistical methods examine changes in outcome measures from before to after the intervention? Were statistical tests done that provided p values for the pre-to-post changes? | No | Yes | No | Yes | C.D. | C.D. | Yes |
| 11. Were outcome measures of interest taken multiple times before the intervention and multiple times after the intervention (i.e., did they use an interrupted time-series design)? | No | Yes | No | Yes | No | Yes | No |
| 12. If the intervention was conducted at a group level (e.g., a whole hospital, a community, etc.) did the statistical analysis take into account the use of individual-level data to determine effects at the group level? | NA | NA | NA | NA | NA | NA | NA |
| Rather 1# | J.A.P | J.A.P | J.A.P | J.A.P | J.A.P | J.A.P | J.A.P |
| Rather 2# | G.Lu | G.Lu | G.Lu | G.Lu | G.Lu | G.Lu | G.Lu |
| Quality rating | Poor | Fair | Poor | Fair | Fair | Poor | Good |

*CD, cannot determine; NA, not applicable; NR, not reported.

|  | Fodstad et al. (1982) | Oliver et al.  (2003) | Rück et al. (2008) | Csigó et al. (2010) | Jung et al. (2014) | Zhan et al.,  (2014) | Batistuzzo et al.  (2015) |
| --- | --- | --- | --- | --- | --- | --- | --- |
| Additional Comments | Reported case studies. Scores of certain tests lack validity to assess cognitive outcome. Not reported p values, but reported significance of the data.  No clear interpretation of cognitive outcomes is made. | There is not detailed information of exclusion and inclusion criteria. | Exclusion/inclusion criteria of the sample was applied post hoc. Radiosurgical devices are not specified. Pre-operative primary rating was obtained retrospectively (n=8). No p values reported in cognitive pre-post changes. | No details of lesion target coordinates. Scores of certain tests lack validity to assess cognitive outcome. | Authors don´t discuss sample size implications or limitations. Exclusion and inclusion criteria may conditioned results (“Significant cognitive impairments (based on a Mini Mental Status Examination, score ⩽ 24”)  Not clarified the score of one test. Not reported p values, but reported significance of the data.  Cognitive outcomes follow-up was “only” of 6 months. | Authors reporter that analysis of psychiatric outcome is made retrospectively. Exclusion and inclusion criteria may conditioned results (“the patient had no cognitive deficit”). Cognitive outcomes assessment is limited. Not reported p values or specific cognitive outcomes data, but reported significance of the data (“cognitive functions showed no significant in post-operative test when compared with pre-operative results” ). | Limited times of assessment influenced by RCT design. |

| Questions | Gong et al. (2018) | Kim et al. (2018) | Peker et al. (2020) | Krámska et al. (2021) | Kassel et al. (2022) | Jung et al. (2006) |
| --- | --- | --- | --- | --- | --- | --- |
| 1. Was the study question or objective clearly stated? | Yes | Yes | Yes | Yes | Yes | Yes |
| 2. Were eligibility/selection criteria for the study population prespecified and clearly described? | Yes | Yes | Yes | Yes | Yes | Yes |
| 3. Were the participants in the study representative of those who would be eligible for the test/service/intervention in the general or clinical population of interest? | Yes | No | No | Yes | Yes | Yes |
| 4. Were all eligible participants that met the prespecified entry criteria enrolled? | No | Yes | C.D. | No | No | Yes |
| 5. Was the sample size sufficiently large to provide confidence in the findings? | Yes | Yes | Yes | Yes | Yes | Yes |
| 6. Was the test/service/intervention clearly described and delivered consistently across the study population? | Yes | Yes | Yes | Yes | Yes | Yes |
| 7. Were the outcome measures prespecified, clearly defined, valid, reliable, and assessed consistently across all study participants? | Yes | Yes | Yes | Yes | Yes | Yes |
| 8. Were the people assessing the outcomes blinded to the participants' exposures/interventions? | No | No | No | No | No | No |
| 9. Was the loss to follow-up after baseline 20% or less? Were those lost to follow-up accounted for in the analysis? | C.D. | Yes | Yes | Yes | Yes | Yes |
| 10. Did the statistical methods examine changes in outcome measures from before to after the intervention? Were statistical tests done that provided p values for the pre-to-post changes? | Yes | Yes | Yes | Yes | Yes | Yes |
| 11. Were outcome measures of interest taken multiple times before the intervention and multiple times after the intervention (i.e., did they use an interrupted time-series design)? | Yes | Yes | No | No | Yes | No |
| 12. If the intervention was conducted at a group level (e.g., a whole hospital, a community, etc.) did the statistical analysis take into account the use of individual-level data to determine effects at the group level? | NA | NA | NA | NA | NA | NA |
| Rather 1# | J.A.P | J.A.P | J.A.P | J.A.P | J.A.P | J.A.P |
| Rather 2# | G.Lu | G.Lu | G.Lu | G.Lu | G.Lu | G.Lu |
| Quality rating | Good | Poor | Poor | Good | Good | Fair |

*CD, cannot determine; NA, not applicable; NR, not reported.

|  | Gong et al. (2018) | Kim et al. (2018) | Peker et al. (2020) | Krámska et al. (2021) | Kassel et al. (2022) | Jung et al.(2006) |
| --- | --- | --- | --- | --- | --- | --- |
| Additional Comments | Data was retrospectively obtained. Included control group for additional comparisons. Different versions of tests are employed. | Exclusion and inclusion criteria may condition results (“Patients who had a significant cognitive impairment (based on a Mini Mental State Examination, score ≤24”). Cognitive outcomes assessment is limited. | Exclusion and inclusion criteria may condition results (“Patients with cognition in the low range”). Data was retrospectively obtained.  Cognitive outcomes assessment is made only at baseline and 6 months after surgery. | Cognitive outcomes assessment is made only at baseline and 6 months after surgery. | Cognitive outcomes assessment is limited. | Data was retrospectively obtained. Refractory definition is fair. Numerical data is not reported regarding cognitive outcomes. |
